# Inhibition of Lysyl Oxidases Impairs Migration and Angiogenic Properties of Tumor-Associated Pericytes

**DOI:** 10.1155/2017/4972078

**Published:** 2017-05-03

**Authors:** Aline Lopes Ribeiro, Carolini Kaid, Patrícia B. G. Silva, Beatriz A. Cortez, Oswaldo Keith Okamoto

**Affiliations:** Centro de Pesquisa sobre o Genoma Humano e Células-Tronco, Departamento de Genética e Biologia Evolutiva, Instituto de Biociências, Universidade de São Paulo, Rua do Matão 277, Cidade Universitária, 05508-090 São Paulo, SP, Brazil

## Abstract

Pericytes are important cellular components of the tumor microenviroment with established roles in angiogenesis and metastasis. These two cancer hallmarks are modulated by enzymes of the LOX family, but thus far, information about LOX relevance in tumor-associated pericytes is lacking. Here, we performed a comparative characterization of normal and tumoral pericytes and report for the first time the modulatory effects of LOX enzymes on activated pericyte properties. Tumoral pericytes isolated from childhood ependymoma and neuroblastoma specimens displayed angiogenic properties in vitro and expressed typical markers, including CD146, NG2, and PDGFR*β*. Expression of all LOX family members could be detected in both normal and tumor-associated pericytes. In most pericyte samples, LOXL3 was the family member displaying the highest transcript levels. Inhibition of LOX/LOXL activity with the inhibitor *β*-aminopropionitrile (*β*APN) significantly reduced migration of pericytes, while proliferation rates were kept unaltered. Formation of tube-like structures in vitro by pericytes was also significantly impaired upon inhibition of LOX/LOXL activity with *β*APN, which induced more prominent effects in tumor-associated pericytes. These findings reveal a novel involvement of the LOX family of enzymes in migration and angiogenic properties of pericytes, with implications in tumor development and in therapeutic targeting tumor microenvironment constituents.

## 1. Introduction

It has been increasingly recognized that the multistep process of tumor development is driven not only by genetic modifications but also by nonmalignant stromal cells within the tumor microenvironment (TME), such as immune cells, endothelial cells, pericytes, and fibroblasts [1, 2]. In addition, extracellular matrix, growth factors, chemokines, and other TME components provide a critical communication network between stromal and tumor cells, impacting both tumor progression and efficacy of anticancer therapies [3–5].

One important, albeit less studied, cellular component of the TME is the pericyte population [6]. Pericytes are present along the microvessel walls where they are important for vasculature remodeling, maturation and stabilization, regulation of blood flow, and blood vessel permeability. Multipotential differentiation capacity [7, 8] as well as immunomodulatory properties, particularly for central nervous system (CNS) pericytes, has also been reported for these cells [9, 10].

Pericytes are closely attached to endothelial cells providing mechanical and physiological support [10]. The endothelial cell-pericyte communication is essential for regulating proliferation, migration, and differentiation of both cell types, which act in concert to coordinate angiogenesis [11]. During the normal vascular sprouting process, pericytes become activated, undergoing phenotypical and functional modifications. Pericyte activation involves enhanced cell migration, proliferation, and secretion of molecules such as proteases, which degrade the basement membrane and modulate the extracellular matrix structure [12, 13].

In cancer, the tumor vascular structure does not achieve proper maturation and, as a consequence, has several abnormalities including irregular pericyte coverage [14]. Furthermore, tumor pericytes are loosely attached to the endothelium and present aberrant cytoplasmic projections invading the tumor parenchyma [13]. It is largely unknown whether mechanisms involved in pericyte activation are affected during tumor angiogenesis.

Recent studies have implicated lysyl oxidases (LOX) in vascular remodeling during angiogenesis [15–17]. This is an emerging role for the LOX family of secreted copper-dependent amine oxidases, whose primary function is the covalent crosslinking of collagens and/or elastin in the extracellular matrix, maintaining tissue strength and structural integrity [18]. The LOX family is comprised of five members: LOX and LOX-like 1–4 (LOXL1–4), which also have important effects on cell senescence, differentiation, and migration [19]. Due to their role in extracellular remodeling, deregulation of enzymes of the LOX family is involved in different diseases [20].

Several studies have extensively demonstrated expression of distinct LOX family members in various types of tumor cells, implicating their functions in tumor progression and metastasis [21, 22]. Despite the growing evidences of LOX contribution to the establishment of a protumorigenic microenvironment [23], there is very few information available about tumor stroma-derived LOX [24, 25]. Thus far, expression of LOX family members in pericytes and LOX influence in pericyte protumorigenic properties have not been examined.

Here, we performed a comparative characterization of normal and tumor-associated pericytes and report for the first time the modulatory effects of LOX enzymes on activated pericyte properties directly implicated in tumor progression, namely migration, proliferation, and angiogenic activity.

## 2. Materials and Methods

### 2.1. Primary Cell Cultures

Pericyte samples, NP-Ad (normal pericytes from adipose tissue) and NP-Mu (normal pericytes from muscle tissue), and mesenchymal stem cells (MSCs), PLAJ3 (adipose tissue) and UC3 (umbilical cord), were obtained, isolated, and cultivated as previously described [26, 27]. Primary cultures of tumor-derived pericytes, TP-Nbl (tumoral pericytes from neuroblastoma) and TP-Epn (tumoral pericytes from ependymoma), were obtained in collaboration with Dr. Hamilton Matushita from the Faculdade de Medicina, Universidade de São Paulo. Isolation and culture methods were conducted as described by Valadares et al. [26]. All procedures were approved by the institutional review board (CEP-IB number 121/2011), and informed consent was obtained from all donors. Human umbilical vein endothelial cells (HUVEC) were purchased directly from ATCC (Manassas, VA, USA) and cultivated under the same conditions as pericytes.

### 2.2. Flow Cytometry

Cells were labeled for 40 minutes at 4°C with the following antibodies: CD146-APC (1 : 100, R&D Systems, Minneapolis, MN, USA); NG2-PE (1 : 100, R&D Systems); PDGFR*β*-PE (1 : 100, R&D Systems); CD31-FITC (1 : 100, BioLegend, San Diego, CA, USA); CD45-APC (1 : 100, BioLegend); CD105-FITC (1 : 100, BioLegend); CD90-PE (1 : 100, BD Biosciences, San Jose, CA, USA); and CD73-PE (1 : 100, BioLegend). Analyses were performed using Guava EasyCyte 5HT™ Flow Cytometer and GuavaSoft 2.1 software (Millipore, Billerica, MA, USA).

### 2.3. In Vitro Angiogenesis Assay and Time-Lapse Analysis

Corning® Matrigel® Basement Membrane Matrix (Corning, Corning, NY, USA) was transferred to a precooled 96-well plate. The plates were incubated at 37°C for 30 minutes. Single cells (2 × 10^4^ cells) were added to each well and incubated in a humidified 5% CO_2_ atmosphere at 37°C. After 2 hours, the tubular networks were examined under the microscope and time-lapse captures were taken every 30 minutes for 24 hours using Incell Analyzer 2200 (GE Healthcare, Little Chalfont, UK). The obtained images were analyzed, and the total amount of lumens formed (considered as internal regions surrounded by cell cords), in three wells per experimental condition, was entirely counted using ImageJ software.

### 2.4. Expression of LOX Family Members

Total RNA was extracted and reverse transcribed as previously described [28]. Reactions of qRT-PCR were performed in triplicate using Power SYBR Green Master Mix (Life Technologies, Carlsbad, CA, USA) on Applied Biosystem 7500 Real-Time PCR System (Life Technologies). Expression of the housekeeping gene *TBP* was used as endogenous control. Quantitative analyses were based on a relative quantification curve, using MSC UC3 as a positive control sample. Primer sequences: TBP-For: GAGCTGTGATGTGAAGTTT CC; TBP-Rev: TCTGGGTTTGATCATTCTGTAG; LOX-For: AAGAGTGAAAAACCAAGG GACA; LOX-Rev: TGGTAGCCATAGTCACAGGATG; LOXL1-For: TG GTAGC CATAGTCACAGGATG; LOXL1-Rev: AAGAGTGAAAAACCAAGGGACA; LOXL2-For: TCGAGGTTGCAGAATCCGATT; LOXL2-Rev: TTCCGTCTCTT CGCTGAAGGA; LOXL3-For: CGGATGTGAAGCCAGGAAAC; LOXL3-Rev: AG GCATCACCAATGTGGCA; LOXL4-For: GGCAGAGTCAGATTTCTCCAACA; LOXL4-Rev: GAGTTCTGCATTGGCTGGGTAT. For detection of LOX/LOXL protein levels, total proteins were extracted with RIPA buffer (Sigma-Aldrich, St. Louis, MO, USA) following the manufacturer's recommendations and submitted to Western blotting standard protocol. Proteins were transferred to nitrocellulose membrane (GE Healthcare). After 1 hour of blocking, membranes were incubated overnight with the following primary antibodies: LOX (Abcam, Cambridge, UK), LOXL3 (Abcam), and GAPDH (Abcam). Blots were incubated for 1 hour with secondary antibody (anti-rabbit HRP-linked) and developed using ECL Chemiluminescence Detection System (GE Healthcare).

### 2.5. Inhibition of LOX/LOXL Activity

*β*-Aminopropionitrile (*β*APN) is commonly used to inhibit the activity of LOX enzymes since it acts as an active site inhibitor [22, 29]. Cells were pretreated with 1 *μ*M *β*APN for 24 hours before the assays. Additionally, *β*APN was also added to the culture medium during all assay procedures at the same final concentration. The inhibition efficacy was verified by measuring H_2_O_2_ after reaction with DCFH-DA (Sigma-Aldrich) in a flow cytometer, as described by Eruslanov and Kusmartsev [30].

### 2.6. Cell Viability Assay

Cell viability was assessed by the MTT assay (Sigma-Aldrich). Briefly, cells at a density of 2 × 10^4^/mL were seeded into a 24-well plate and incubated at 37°C with 5% CO_2_ humidified atmosphere for 24 hours. Cells were treated for 24, 48, and 72 hours with fresh medium containing 1 *μ*M *β*APN or vehicle (control). After treatment periods, MTT solution was added at concentration of 166.67 *μ*g/mL and incubated for 4 hours at 37°C. The supernatant was discarded and formazan crystals were dissolved with DMSO. The optical density was measured at 550 nm using Epoch Microplate Spectrophotometer (BioTek, Winooski, VT, USA).

### 2.7. Cell Migration Assay

Cell migration assay was performed using modified Boyden chambers as described by Chen [31]. Briefly, 1 × 10^5^ cells were plated at the upper compartment of a Transwell® with 8.0 *μ*m pore polyester membrane insert (Corning) and medium with 10% FBS was placed at the inferior compartment to act as a chemoattractive solution. Following an incubation period (3 hours for tumor pericytes and 12 hours for normal tissue pericytes), the cells that migrated through the membrane were fixed and stained with 1% of toluidine blue and 1% borax solution. Stained cells were lysed with 1% SDS, and optical density was measured at 626 nm.

### 2.8. EdU Incorporation Assay

Cells in the S phase of cell cycle were determined by EdU incorporation assay using Click-It EdU Alexa Fluor 488 Imaging Kit (Life Technologies) and following the manufacturer's recommendation. EdU (10 *μ*M) incorporation time was 2 hours for all pericyte cultures.

### 2.9. Statistical Analysis

Data were analyzed by ANOVA followed by the Bonferroni post hoc test or unpaired Student's *t*-test (GraphPad Prism 5 software, GraphPad Software, La Jolla, CA, USA) as indicated in the figure legends. All experiments were performed at least in triplicate, and three independent experiments were carried out. Results are presented as mean ± SD.

## 3. Results

### 3.1. Abnormal Tumoral Pericyte Properties

**Figure 1:**
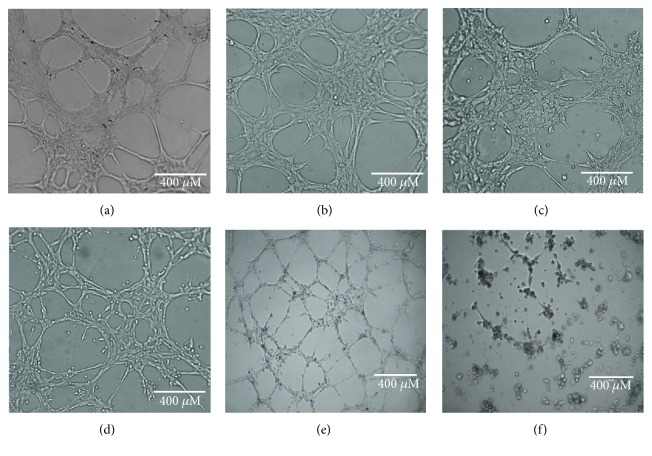
Angiogenic properties of pericytes. Both normal (NP-Ad, NP-Mu) and tumor-associated pericytes (TP-Nbl, TP-Epn) were capable of forming tube-like structures in vitro, generating interconnected networks similar to those generated by endothelial cells (HUVEC). Mesenchymal stem cells (MSCs) do not share this propriety and, therefore, were not able to generate such tube-like structures under the same experimental conditions. (a) NP-Ad; (b) NP-Mu; (c) TP-Nbl; (d) TP-Epn; (e) HUVEC; (f) MSC.

As indicated in Table 1, tumor-associated pericytes displayed a rather heterogeneous pattern of expression of the typical pericyte markers, CD146, NG2, and PDGFR*β*, compared with normal pericytes. However, as expected, both normal and tumor-associated pericyte populations were mainly negative for the expression of endothelial and hematopoietic cell markers and did not display the combined expression of CD90, CD73, and CD105, a required characteristic of mesenchymal stromal cells (MSCs) [32]. Further functional tube formation assays confirmed that all normal and tumor-associated pericyte cultures were comprised by cells capable of generating capillary-like structures in vitro (Figures 1(a), 1(b), 1(c), and 1(d)), a characteristic property of pericytes and endothelial cells, but not of MSCs (Figures 1(e) and 1(f)).

**Figure 2:**
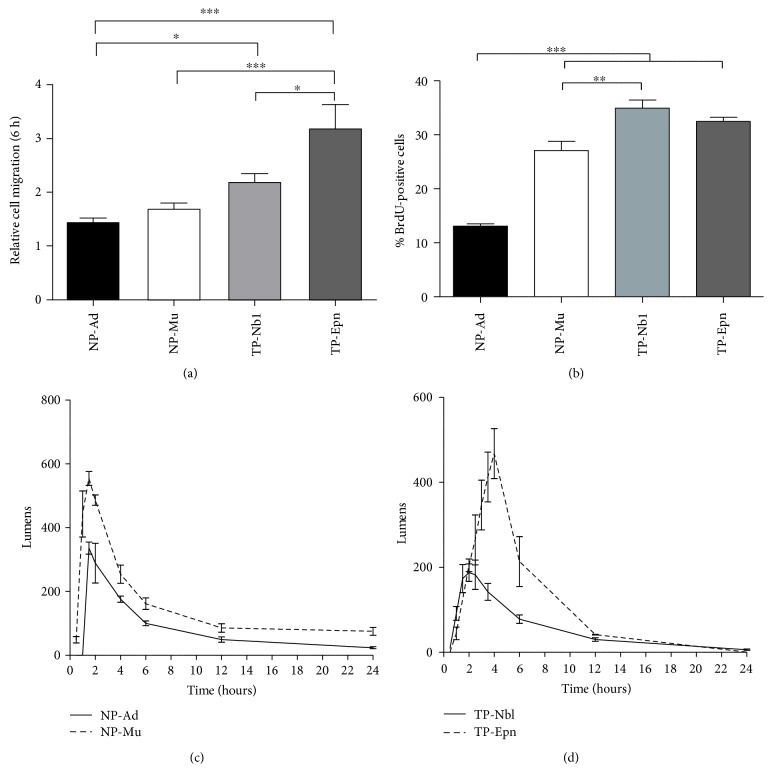
Activated properties of normal and tumor-associated pericytes. (a) Cell migration was calculated after 6 h in the presence of chemical stimulus, relative to basal cell migration without chemotaxis. (b) Cell proliferation based on BrdU incorporation after 24 h in culture. Kinetics of tube-like formation by (c) normal pericytes and (d) tumor-associated pericytes, under angiogenic conditions in vitro. Normal pericyte samples: NP-Ad and NP-Mu. Tumor-associated pericyte samples: TP-Nbl and TP-Epn. Significance level: ^∗^*P* < 0.05, ^∗∗^*P* < 0.01, and ^∗∗∗^*P* < 0.001.

A more detailed analysis revealed that, under the same experimental conditions, tumor-associated pericytes presented significantly higher migration and proliferation rates compared with normal pericytes (Figures 2(a) and 2(b)). When comparing the kinetics of tube formation in vitro (Figures 2(c) and 2(d)), tumor-associated pericytes either generated a lower amount of total capillary-like structures or took a longer time to attain the maximum amount of such structures than normal pericytes, indicating an abnormal in vitro behavior.

### 3.2. Expression Profile of Lysyl Oxidases in Pericytes

Since no previous studies were available regarding expression of LOX family members in pericytes, a LOX/LOXL expression profiling was carried out in the two normal tissue-derived pericytes and two tumor-associated pericytes and compared with the expression levels detected in MSCs and endothelial cells, which are known to be positive for LOX/LOXL expression.

**Figure 3:**
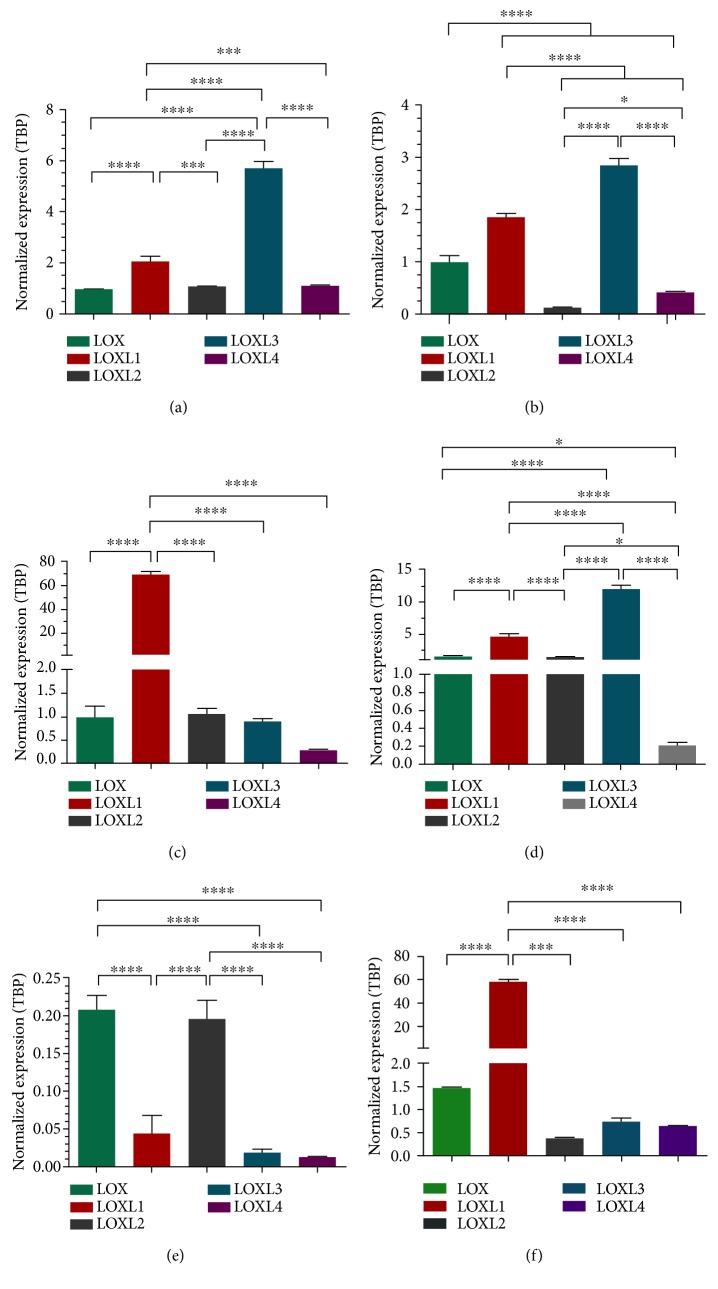
Comparative analysis of LOX and LOXL1–4 expression profiles in normal and tumor-associated pericytes. Gene expression was assessed by quantitative real-time PCR, using *TBP* as endogenous control. Normal pericyte samples: NP-Ad and NP-Mu. Tumor-associated pericyte samples: TP-Nbl and TP-Epn. Endothelial cells (HUVEC) and mesenchymal stem cells (MSCs) were also included in the analysis as control samples. (a) NP-Ad; (b) NP-Mu; (c) TP-Nbl; (d) TP-Epn; (e) HUVEC; (f) MSC. Significance level: ^∗^*P* < 0.05, ^∗∗^*P* < 0.01, ^∗∗∗^*P* < 0.001, and ^∗∗∗∗^*P* < 0.0001.

At the transcriptional level, a concordant LOX/LOXL expression pattern was detected in the two normal tissue-derived pericytes analyzed. Expression of all LOX family members could be detected in these cells, with LOXL3 exhibiting the highest expression levels (Figures 3(a) and 3(b)). A similar LOX/LOXL expression pattern was detected in the pericyte sample derived from ependymoma, but not in the other pericyte sample derived from neuroblastoma (Figures 3(c) and 3(d)). In the latter case, LOXL1 displayed the highest expression levels, such as the one exhibited by MSCs (Figure 3(f)).

Nonetheless, the LOX/LOXL expression profile detected in normal and tumor-associated pericytes differed from the one exhibited by endothelial cells, in which LOX and LOXL2 are the predominantly expressed family members (Figure 3(e)).

**Figure 4:**
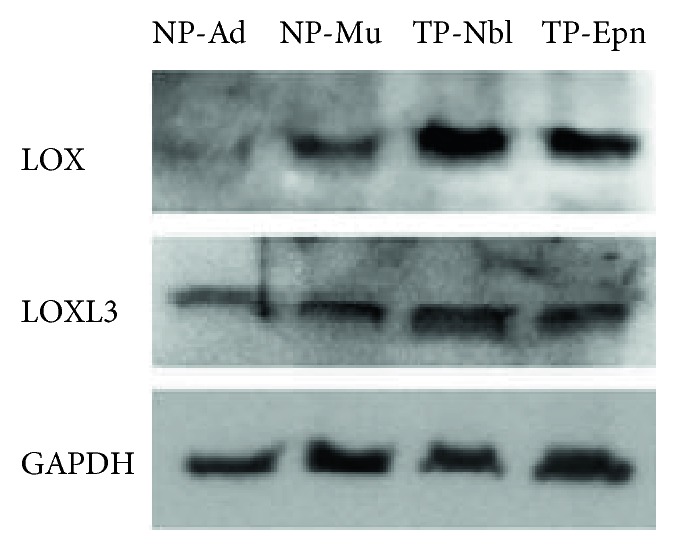
LOX and LOXL3 protein levels in normal and tumor-associated pericytes. Representative Western blots for each protein are shown. Normal pericyte samples: NP-Ad and NP-Mu. Tumor-associated pericyte samples: TP-Nbl and TP-Epn.

Expression of LOX and LOXL3 in pericytes was also confirmed at the protein level. Higher LOX levels were detected in tumor-associated pericytes compared with normal pericytes (Figure 4).

### 3.3. Inhibition of Lysyl Oxidases Affects Protumorigenic Properties of Pericytes

The effects of LOX/LOXL on activated pericyte properties were investigated using the specific inhibitor *β*APN, under experimental conditions ensuring significant inhibition of enzymatic activity, without affecting cell viability (Supplementary Figure available online at https://doi.org/10.1155/2017/4972078).

**Figure 5:**
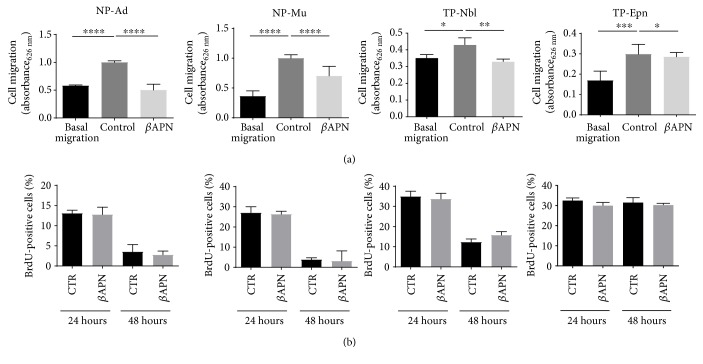
Effects of *β*APN on migration and proliferation of normal and tumor-associated pericytes. (a) Cell migration measured after 24 h treatment with 1 *μ*M *β*APN. (b) Cell proliferation based on BrdU incorporation after 24 h and 48 h treatment with 1 *μ*M *β*APN. Normal pericyte samples: NP-Ad and NP-Mu. Tumor-associated pericyte samples: TP-Nbl and TP-Epn. Significance level: ^∗^*P* < 0.05, ^∗∗^*P* < 0.01, ^∗∗∗^*P* < 0.001, and ^∗∗∗∗^*P* < 0.0001.

Upon inhibition of LOX/LOXL activity, a significant reduction in migration of both normal and tumor-associated pericytes was detected. Cell proliferation rates, however, were not altered under the same experimental conditions (Figures 5(a) and 5(b)).

**Figure 6:**
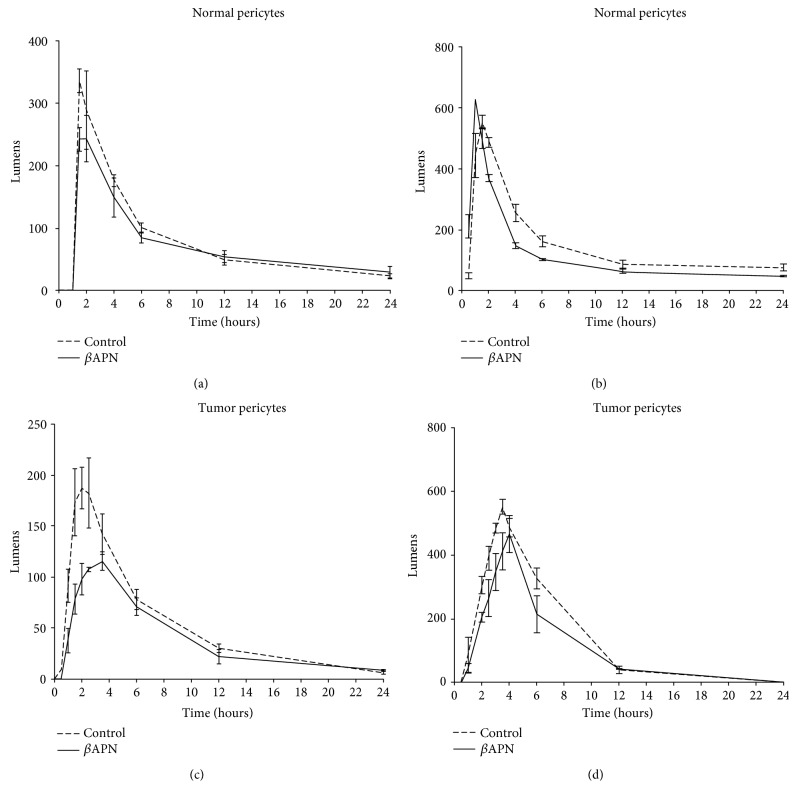
Effects of *β*APN on angiogenic properties of normal and tumor-associated pericytes. The kinetics of tube-like formation by pericytes was assessed for 24 hours under treatment with 1 *μ*M *β*APN in vitro. Normal pericyte samples: NP-Ad and NP-Mu. Tumor-associated pericyte samples: TP-Nbl and TP-Epn. (a) NP-Ad; (b) NP-Mu; (c) TP-Nbl; (d) TP-Epn.

Tube formation dynamics in vitro by pericytes was also affected upon inhibition of LOX/LOXL activity (Figure 6). Compared with control conditions, there was a significant reduction in the total number of lumens formed and, for some pericyte cultures, the kinetic curves were also slightly dislocated. The most prominent effects were observed in cultures of tumor-associated pericytes, where the time for reaching the maximum amount of capillary-like structures was longer after inhibition of LOX/LOXL activity.

Altogether, these results indicate that blocking activity of LOX family members may influence pericyte properties that are essential to their protumorigenic roles within the TME.

## 4. Discussion

Understanding pericyte properties is essential given the effects that these cells may exert within the TME [33–37]. Despite the lack of consensus in the current literature about definite criteria for pericyte identification, the selected normal and tumor-derived pericytes used in this study displayed in vitro angiogenic properties and expression of cellular markers typically reported for this type of cell [38]. Notably, expression of CD146, NG2, and PDGFR*β* was somewhat reduced in the population of tumor-associated pericytes, in agreement with previous studies reporting variable expression of some of these markers in pathological conditions. In perivascular soft tissue tumors, a reduced expression of *α*SMA, CD146, and PDGFR*β* in pericytes from malignant glomus tumors has been implicated in partial loss of pericytic differentiation [39]. In brain tumors, low expression of the NG2 proteoglycan in pericytes has been reported to affect their interaction with endothelial cells and disturb tumor vasculogenesis [40].

Within the CNS, pericytes play key physiological roles with effects in brain angioarchitecture, blood-brain barrier, and neuroinflammation [41–43]. Pericytes may also affect tumor malignancy and clinical prognosis, particularly in CNS tumors that are highly vascularized [44, 45]. In the present study, the increased migration and proliferation rates observed for tumor-associated pericytes compared with normal pericytes are consistent with the TME context. Tumor stromal cells, including fibroblasts and endothelial cells, are known to be constantly activated, exhibiting enhanced migration and proliferative behavior [46, 47]. Mechanisms underlying tumoral pericyte activation are, however, still elusive.

A growing body of evidences indicates that processes relevant to tumor progression, such as angiogenesis and metastasis, are modulated by enzymes of the LOX family [21, 23, 48]. Although these two processes involve interaction with the tumor stroma, thus far, very little is known about LOX expression in cellular components of the TME, and data on LOX expression in pericytes is lacking. Here, both normal and tumor-derived pericytes were found to express all members of the LOX family of enzymes. At the transcript level, the pattern of LOX/LOXL expression observed was very similar among the pericyte samples, the exception being the neuroblastoma-derived pericytes in which LOXL1 was more highly expressed than the other family members.

Apart from its role in extracellular matrix modulation and implications in pathogenesis [49, 50], no other functions are known for LOXL3, the family member that displayed the highest expression level in most pericyte samples. On the other hand, expression of LOX and LOXL2 has been frequently correlated with all stages of tumorigenesis. Secretion of LOXL2 by both stromal and tumor cells, for instance, is involved in fibroblast activation within the TME [51, 52]. Regarding LOX/LOXL expression in tumor stromal cells, a recent study with a mouse model of mammary tumor progression showed that LOX expressed by carcinoma-associated fibroblasts stimulates tumor cell intravasation into the vasculature and ensuing metastasis [23].

The significant inhibition of migration and angiogenic properties of pericytes detected upon inhibition of LOX/LOXL activity with the inhibitor *β*APN is in agreement with a previous study reporting inhibition of tumor angiogenesis by *β*APN treatment in vivo [53]. Since knockdown of *LOX* and *LOXL2* has also been reported to inhibit motility and network-forming ability of endothelial cells [49], expression of LOX/LOXL in both pericytes and endothelial cells is likely indispensable for proper angiogenesis. Interestingly, in the in vitro angiogenesis assays, the inhibitory effects of *β*APN treatment were more pronounced in tumor-associated pericytes than in their normal counterparts, given that not only a lower amount of total tube-like structures was observed but also a longer time to achieve the peak of such structures. This result is in concert with the higher LOX protein levels detected in tumor-associated pericytes, encouraging preclinical studies addressing whether antiangiogenic approaches based on LOX/LOXL inhibitors may be designed to be preferentially harming to pericytes associated with the tumor vasculature.

## 5. Conclusions

In summary, our findings reveal a novel involvement of LOX/LOXL family of enzymes in migration and angiogenic properties of both normal and tumor-associated pericytes, supporting the exploration of LOX/LOXL inhibitors to target activation of pericytes within the TME.

## Supplementary Material

Supplementary Figure 1. Inhibition of LOX / LOXL activity in human pericytes (NP-Mu), after 24h incubation with 1μM βAPN. Significance level: ∗P<0.05. Supplementary Figure 2. Cell viability after 24h, 48h, and 72h treatment with 1μM βAPN. Normal pericyte samples: NP-Ad, NP-Mu. Tumor-associated pericyte samples: TP-Nbl, TP-Epn. (A) NP-Ad; (B) NP-Mu; (C) TP-Nbl and (D) TP-Epn. Significance level: ∗P<0.05, ∗∗P<0.01.

## Figures and Tables

**Table 1:** Expression of cell surface markers in pericytes derived from normal tissue and pediatric nervous system tumors.

Cell surface marker	Normal pericytes		Tumor-associated pericytes	
	NP-Ad (%)^∗^	NP-Mu (%)^∗^	TP-Nbl (%)^∗^	TP-Epn (%)^∗^
CD146	95,0	65,5	0,4	46,4
NG2	99,4	94,1	22,7	99,6
PDGFR*β*	93,1	70,3	63,0	59,6
CD31	0,3	0,8	1,0	10,2
CD45	0,8	0,2	0,1	0,1
CD105	5,0	11,7	2,2	7,2
CD90	34,1	60,6	24,6	99,7
CD73	100,0	100,0	100,0	100,0

^∗^Percentage of positive cells for each surface marker, within a given pericyte culture, as determined by flow cytometric analysis. NP-Ad: normal pericytes from adipose tissue; NP-Mu: normal pericytes from muscle tissue; TP-Nbl: tumoral pericytes from neuroblastoma; TP-Epn: tumoral pericytes from ependymoma.
