# Supplementary material for: Inhibition of Lysyl Oxidases Impairs Migration and Angiogenic Properties of Tumor-Associated Pericytes
Source: Stem Cells Int. 2017 May 3;2017:4972078. doi: 10.1155/2017/4972078 (PMC5434472; doi:10.1155/2017/4972078)
Supplement: Supplementary file 1 — Supplementary Figure 1. Inhibition of LOX / LOXL activity in human pericytes (NP-Mu), after 24h incubation with 1μM βAPN. Significance level: ∗P<0.05. Supplementary Figure 2. Cell viability after 24h, 48h, and 72h treatment with 1μM βAPN. Normal pericyte samples: NP-Ad, NP-Mu. Tumor-associated pericyte samples: TP-Nbl, TP-Epn. (A) NP-Ad; (B) NP-Mu; (C) TP-Nbl and (D) TP-Epn. Significance level: ∗P<0.05, ∗∗P<0.01. [file 4972078.f1.docx]

**Supplementary Figures**

**Supplementary Figure 1.** Inhibition of LOX / LOXL activity in human pericytes (NP-Mu), after 24h incubation with 1µM βAPN. Significance level: *P < 0.05.


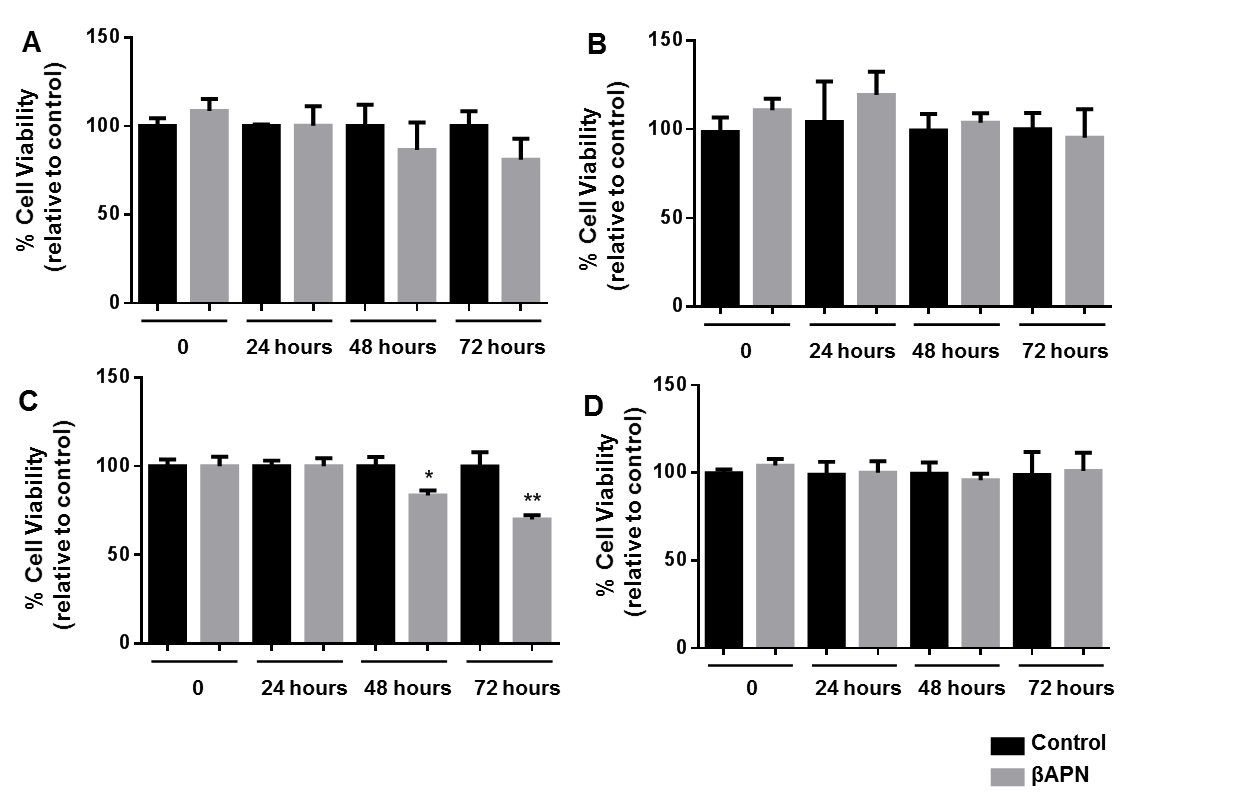


**Supplementary Figure 2.** Cell viability after 24h, 48h, and 72h treatment with 1µM βAPN. Normal pericyte samples: NP-Ad, NP-Mu. Tumor-associated pericyte samples: TP-Nbl, TP-Epn. (A) NP-Ad; (B) NP-Mu; (C) TP-Nbl and (D) TP-Epn. Significance level: *P < 0.05, **P < 0.01.
